# Factors associated with caregiver burden among adult (19–64 years) informal caregivers – An analysis from Dutch Municipal Health Service data

**DOI:** 10.1111/hsc.12982

**Published:** 2020-03-23

**Authors:** Emma Koopman, Monique Heemskerk, Allard J. van der Beek, Pieter Coenen

**Affiliations:** ^1^ Public Health Service Zaanstreek‐Waterland Zaanstad The Netherlands; ^2^ Department of Public and Occupational Health Amsterdam Public Health Research Institute Vrije Universiteit Amsterdam Amsterdam The Netherlands

**Keywords:** cross‐sectional, health survey, informal care, informal caregivers, perceived burden

## Abstract

Due to the ageing population and the rising prevalence of chronic diseases, it is expected that the demand on informal caregivers will increase. Many informal caregivers experience burden, which can have negative consequences for their own health and that of the care recipient. To prevent caregiver burden, it is important to investigate factors associated with this burden. We aimed to identify factors associated with caregiver burden in adult informal caregivers. Among a sample of adult informal caregivers (*n* = 1,100) of the Dutch region of Zaanstreek‐Waterland, perceived caregiver burden, demographic factors, caregiving situation, health‐related factors and socio‐financial factors were measured as part of the national Health Survey in 2016. Using univariate and multivariate logistic regression analysis, for which a backward selection method was applied, associations with caregiver burden were studied. In the multivariate model, time spent providing informal care was significantly associated with perceived caregiver burden, with an odds ratio (OR) [95% confidence interval] of 7.52 [3.93–14.39] for those spending >16 hr compared to 1–2 hr on informal care. Also providing care to their child(ren) (OR: 2.55 [1.51–4.31]), poor perceived health (OR: 1.80 [1.20–2.68]) and loneliness of the caregiver (OR: 2.05 [1.41–2.99]) were significantly associated with caregiver burden. To possibly prevent and reduce informal caregiver burden, factors associated with such burden should be intervened on. As such, special attention should be paid to caregivers who provide many hours of care or provide care to their child(ren), as well as those who have a poor perceived health themselves and/or experience feelings of loneliness.

**What is known about this topic**

- Demands on informal caregivers are likely to increase as a result of the ageing population and increasing prevalence of chronic disease.
- Informal caregiver burden can have negative consequences for caregiver's own health and that of their care recipient.
- Up to date information on factors associated with caregiver burden in the general adult population is scarce.

**What this paper adds**

- We identified factors associated with caregiver burden in Dutch adult informal caregivers.
- We found that time spent providing informal care, providing care to child(ren), poor perceived health and loneliness were significantly associated with perceived caregiver burden.
- These factors should be intervened on in order to prevent and reduce informal caregiver burden.

## 1 INTRODUCTION

An increase in the average age expectancy and chronic diseases, such as cardiovascular disease and cancer, caused a substantial rise in the demand for healthcare over the last decades (Marengoni et al., 2011; Prevo et al., 2018). Improvements in treatment have resulted in a higher survival rate and hospitalisation (Piran, Khademi, Tayari, & Mansouri, 2017). However, due to budget cuts and political choices, there is less space for long‐term formal patient care (Deeken, Taylor, Mangan, Yabroff, & Ingham, 2003), as many local and national governments aim to decentralise care provision (Eurocarers, 2017). As such, the responsibility for long‐term care has partly shifted from healthcare facilities to family and friends of patients, which has resulted in a growing need for these informal caregivers to maintain a sustainable healthcare system (Arno, Levine, & Memmott, 1999; Deeken et al., 2003). In the Netherlands, responsibility for supporting and guiding informal caregivers has been allocated to municipalities, and many governments of other countries also aim to decentralise care provision (Eurocarers, 2017). This policy change is likely to have important effects on the burden of informal caregivers (Kelders et al., 2016). In the Netherlands it was shown that 14% of the population (i.e. those aged ≥19 years) provided informal care in 2016, of whom 14% feel ‘heavily burdened’ as a consequence of providing this care (Statistics Netherlands, 2017).

Caregiver burden has been defined as the distress informal caregivers experience as a result of providing care, influenced by characteristics of the patient, the individual caregiver and the environment (Grant et al., 2013). Previous studies have reported that a perceived high burden among informal caregivers can have negative consequences for their own mental and physical health (Cuijpers, 2005; Schulz & Beach, 1999; Schulz, O'Brien, Bookwala, & Fleissner, 1995; Shiba, Kondo, & Kondo, 2016), as it has been associated with depression (Schoenmakers, Buntinx, & Delepeleire, 2010), stress (Vitaliano, Zhang, & Scanlan, 2003), and low self‐efficacy and well‐being (Pinquart & Sorensen, 2003). Moreover burdened informal caregivers are often impaired in their caregiving, which could have an impact on the health of the care recipient (Shiba et al., 2016).

Previous studies have identified a number of factors associated with burden among those who provide informal care to people with a chronic illness (Karakis et al., 2014; Pinquart & Sorensen, 2011; Vaingankar, Subramaniam, Abdin, He, & Chong, 2012). This includes demographics (e.g. gender, age, marital status, educational level, employment status and household composition), caregiving situation (e.g. the number of hours spent at providing care and the type of relationship with the care recipient), health‐related factors (e.g. perceived health, chronic diseases and mental health) and socio‐financial factors (e.g. loneliness, financial difficulties, mastery of life, contact with neighbours and social cohesion in neighbourhood). However, information on factors associated with caregiver burden in the general adult population, in particular from data collected after recent policy changes, is scarce.

The present cross‐sectional study aims to investigate which factors are associated with high‐perceived caregiver burden of adult (19–64 years) informal caregivers. In order to do so, we studied a range of factors in a large sample of adults from a population‐based cohort of adult inhabitants of Zaanstreek‐Waterland, the Netherlands. Insights from this study might guide policy and interventions aimed at prevention of caregiver burden.

## 2 METHODS

### 2.1 Study design and participants

Conducting and reporting of this study were done in accordance with the *Strengthening the Reporting of Observational Studies in Epidemiology* (STROBE) statement guidelines (von Elm et al., 2007). The medical ethical committee of Amsterdam UMC, location AMC has declared that the Dutch Medical Research Involving Human Subjects Act (WMO) does not apply to this study and that official approval of this study is not required by the medical ethical committee is thus not required. This study was performed using cross‐sectional data, gathered by the *Health Survey* of the Public Health Services of Zaanstreek‐Waterland and Statistics Netherlands. The Health Survey is a nationwide survey conducted by Dutch Public Health Services in collaboration with the National Institute for Public Health and the Environment (RIVM) and Statistics Netherlands, with the purpose to get insight into the health of citizens on a local, regional and national level. The Health Survey is repeated every 4 years. For the Health Survey in Zaanstreek‐Waterland (a region existing of eight municipalities north of Amsterdam, the Netherlands), a random sample of adult (19–64 years) citizens in the Zaanstreek‐Waterland area received an information letter as well as a hardcopy version of the questionnaire and a code to access the digital version of that same questionnaire in the autumn of 2016. While a comparable questionnaire was sent out to older citizens (>64 years), there were some important differences in the questions asked making it difficult to combine these data with those from the adult questionnaire. Therefore, only participants aged 19–64 years and providing informal care are included in the present study.

### 2.2 Measures

Self‐reported data on informal care and caregiver burden, as well as associated factors and descriptive variables were obtained. The list of associated factors was composed based on the apparent association with caregiver burden as obtained from the literature (Karakis et al., 2014; Pinquart & Sorensen, 2011; Vaingankar et al., 2012).

#### 2.2.1 Informal care and caregiver burden

According to the Dutch Public Health Service and RIVM (RIVM, 2016), informal caregiving is defined as *the unpaid nonprofessional care that people provide to a person from his or her social network, such as a partner, child or friend, if this person is sick, disabled and needs help for a longer period*. Here, ‘a longer period’ is defined as providing care for at least 3 months and 8 hr per week (Statistics Netherlands, 2017). Participants were asked whether they were providing informal care in the past 12 months (yes/no), if they provided informal care right now (yes/no), for how many hours they were providing informal care per week and for how long they provided care (at least 3 months or less). Only participants who were currently providing informal care for a ‘longer period’ were included for further analyses.

The main dependent variable, perceived caregiver burden, was measured according to methods of Statistics Netherlands and the Dutch Public Health Services (RIVM, 2016), using a 5‐point Likert scale (Allen & Seaman, 2007). Informal caregivers who reported that they were ‘not (really) burdened’ or ‘somewhat burdened’ were defined as not burdened. Informal caregivers who filled in that they were ‘quite heavily burdened’, ‘very heavily burdened’ or ‘overburdened’ were defined as burdened. This dichotomised variable was used in the analysis.

#### 2.2.2 Demographic factors

The following demographic factors were collected: gender (male/female), age (categorised into 19–34, 35–49 and 50–64 years), marital status (dichotomised into living together with a partner vs. living alone). Moreover the highest completed level of education was assessed according to the Standard Education Classification of Statistics Netherlands (i.e. primary, secondary and tertiary level education). Employment status was assessed by asking participants whether they have paid work, and if so, for how many hours per week. Responses were dichotomised into ≥20 hr/week of paid work and <20 hr/week of paid work or being unemployed. Finally, household composition was questioned with outcome categories dichotomised into single parent families (living together with child(ren), but not with their partner, parent(s) or other adult(s)) versus non‐single parent families.

#### 2.2.3 Caregiving situation factors

Caregiving situation was characterised by time (in hours) invested in providing informal care and the type of relationship between the informal caregiver and the care recipient. Participant's average time invested in providing informal care (including travel time) was categorised into 1–2, 3–4, 5–8, 9–16 and >16 hr per week. The informal caregiver's self‐reported relationship with their care recipient was dichotomised into informal caregivers who provided care to their child(ren) (in law) versus recipients with other relationship types (spouse/partner, parents (in law), other family members and neighbours, friends and acquaintances). This categorisation was made since caregivers who provide care to their child(ren) (in law) stood out with respect of the other categories in terms of their caregiver burden (Table 1). Informal caregivers who provided care to more than one care recipient were excluded, to be able to assess the independent association of the relationship between informal caregivers and their recipient on caregiver burden. However, there were no clear differences in participant characteristics and caregiver burden between informal caregivers who provided informal care to either one or more than one care recipients (data not reported in this paper).

**TABLE 1:** Characteristics of the study population (column 1), the informal caregivers (column 2) and the burdened informal caregivers (column 3). The study sample comprised 8,544 inhabitants of ZW (column 1), of whom approximately 16% were informal caregivers (column 2) and 15% of these informal caregivers were burdened (column 3)

	(1) Total study population 100% *n* = 8,544	(2) Informal caregivers 16.2% of (1) *n* = 1,289	(3) Burdened informal caregivers^a^ 15.2% of (2) *n* = 196
	*n* (%)	*n* (%)	*n* (%)
*Demographic factors*			
Gender			
Male	3,672 (43)	395 (31)	59 (30)
Female	4,872 (57)	894 (69)	137 (70)
Age			
19–34 year	1,853 (22)	93 (7)	15 (8)
35–49 year	2,839 (33)	314 (24)	56 (29)
50–64 year	3,852 (45)	882 (68)	125 (64)
Marital status			
Not married/partnered	2,387 (29)	260 (21)	43 (23)
Married/partnered	5,994 (72)	1,002 (79)	148 (78)
Educational level			
Primary	2,045 (27)	336 (27)	45 (24)
Secondary	3,143 (41)	499 (40)	79 (42)
Tertiary	2,536 (33)	410 (33)	64 (34)
Employment status			
Not ≥20 hr of paid work	2,417 (31)	497 (40)	81 (43)
≥20 hr of paid work	5,298 (69)	748 (60)	108 (57)
Household composition			
No single‐parent family	7,620 (91)	1,163 (92)	171 (90)
Single‐parent family	790 (9)	104 (8)	20 (11)
*Caregiving situation*			
Hours spent providing care	n/a		
1–2 hr		413 (33)	21 (11)
3–4 hr		310 (25)	31 (16)
5–8 hr		248 (20)	37 (19)
9–16 hr		174 (14)	57 (30)
>16 hr		118 (9)	46 (24)
Relationship to care recipient	n/a		
Not child(ren)^b^		999 (91)	130 (77)
Child(ren)^b^		101 (9)	38 (23)
Relationship to care recipient			
Not spouse^b^		1,008 (92)	148 (88)
Spouse^b^		92 (8)	20 (12)
Relationship to care recipient			
Not parent(s)^b^		388 (35)	74 (44)
Parent(s)^b^		712 (65)	94 (56)
*Health‐related*			
Perceived health			
Moderate, poor or very poor	1,762 (21)	280 (22)	75 (38)
(Very) good	6,766 (79)	1,008 (78)	121 (62)
Chronic disease			
No chronic disease	5,756 (68)	793 (62)	97 (50)
Chronic disease	2,758 (32)	490 (38)	98 (50)
Mental health			
No high risk of anxiety or depression	7,747 (94)	1,193 (94)	162 (84)
High risk of anxiety or depression	534 (6)	75 (6)	30 (16)
*Socio‐financial*			
Loneliness			
Not lonely	4,852 (62)	789 (63)	87 (46)
Moderate to severe loneliness	2,986 (38)	460 (37)	103 (54)
Financial difficulties			
No financial difficulties	6,271 (81)	1,007 (81)	131 (70)
Some/great financial difficulties	1,445 (19)	234 (19)	56 (30)
Mastery of life			
Insufficient	688 (9)	112 (9)	42 (23)
Moderate	5,420 (68)	849 (69)	118 (63)
High	1,877 (24)	278 (22)	26 (14)
Contact neighbours			
Weekly or more	5,509 (70)	974 (77)	143 (75)
Less than weekly	2,311 (30)	286 ( 23)	49 (26)
Social cohesion neighbourhood			
Getting (very) well along together	5,409 (70)	885 (70)	115 (61)
Not getting (very) well along together	2,368 (30)	372 (30)	75 (39)

Note

Number of respondents might vary between variables due to item non‐response

a Burdened informal care givers are defined as informal caregivers who filled in that they were ‘quite heavily burdened’, ‘very heavily burdened’ or ‘overburdened’.

b Cases where informal caregivers filled in more than one option for care recipient type were not taken into regression analysis.

#### 2.2.4 Health‐related factors

Regarding health‐related factors, perceived health, having chronic diseases and mental health were assessed. Participants were asked to rate their perceived health, using a 5‐point Likert scale (Oldenkamp, Wittek, Hagedoorn, Stolk, & Smidt, 2016) ranging from ‘very poor health’ (1) to ‘very good health’ (5). The highest two categories were defined as good perceived health, and the other three categories as poor perceived health. Participants were also asked whether they had any chronic disorder(s) (yes/no). To assess mental disorders, the Kessler Psychological Distress Scale (K10; Kessler et al., 2003) was used to assess anxiety and depressive symptoms in the past 4 weeks. The K10 is a 10‐item questionnaire and summary scores were dichotomised into low or moderate risk (10–29) and high risk (30–50) of anxiety and depression (Andrews & Slade, 2001; Kessler et al., 2002).

#### 2.2.5 Socio‐financial factors

The 11 statements defined by Jong‐Gierveld were used to measure loneliness, with participants responding by ‘yes’ (1), ‘more or less’ (0) or ‘no’ (0). Summary scores were dichotomised into not lonely (0–2) and lonely (3–11; de Jong Gierveld & van Tilburg, 2010). Participants were also questioned about having financial difficulties by asking whether they had difficulties in getting by. Participants who filled in ‘not at all’ or ‘no difficulties but have to pay attention to expenses’ were categorised as having no financial difficulties, while participants who filled in that they had ‘some difficulties’ or ‘great difficulties’ were categorised as having financial difficulties. The Pearlin Mastery Scale (PM; Pearlin & Schooler, 1978) was used to measure an individual's level of mastery, which is the extent to which someone regards his/her life chances as being under his/her own control. The PM is a 7‐item scale including five negatively worded items and two positively worded items with response options from ‘strongly disagree’ to ‘strongly agree’, resulting in a summary scores ranging from 7 to 28. Summary scores were categorised into insufficient (7–19), moderate (20–31) and high level (32–35) of mastery. Participants also reported how often they had contact with their neighbours, with outcome categories dichotomised into ‘weekly or more’ versus ‘less than weekly’ (monthly, less than a month and almost never). To measure the perceived social cohesion in the neighbourhood, participants also responded to the statement ‘people in my neighbourhood are not getting (very) well along’, using a 5‐point Likert scale, in which the first three and last two response options were combined to derive at a dichotomous categorisation.

### 2.3 Statistical analysis

Of all variables, descriptive statistics were provided. Logistic regression was used to assess the association of aforementioned sociodemographic, caregiver situation, health‐related and socio‐financial factors, that have shown to be associated with caregiver burden in previous research (Buchanan, Radin, & Huang, 2011; Karakis et al., 2014; Pinquart & Sorensen, 2011; Vaingankar et al., 2012), with caregiver burden. We developed a prediction model using three steps, according to earlier work (Huysmans et al., 2017). In the first step, a univariate analysis was applied to examine the association between each of the aforementioned variables and caregiver burden individually. Variables associated with caregiver burden with a conservative level of significance of *p* < .2 were retained. In the second step, a backward stepwise regression analysis was applied within each of the four categories (i.e. sociodemographic, caregiver situation, health‐related and socio‐financial) of factors, retaining variables being associated with caregiver burden (with level of significance *p* < .1). In the third step, all remaining variables from step two were analysed together, again using a backward stepwise regression analysis with removal set at *p* < .1 (Tran, Arabnia, Mohammed, Naugler, & Far, 2015). Prior to step two and three, correlation of all variables were calculated. In cases where two variables were strongly correlated (with a Spearman *r* ≥ .7), only the variable with the largest standard deviation was retained to avoid multi‐collinearity.

Effects were presented in odds ratios (OR) with 95% confidence intervals (95% CI). Goodness‐of‐fit was calculated by Nagelkerke's *R* ^2^ to evaluate explained variance of the final model. All statistical analyses were performed using SPSS (IBM Statistics version 24).

## 3 RESULTS

### 3.1 Sample characteristics

**FIGURE 1:**
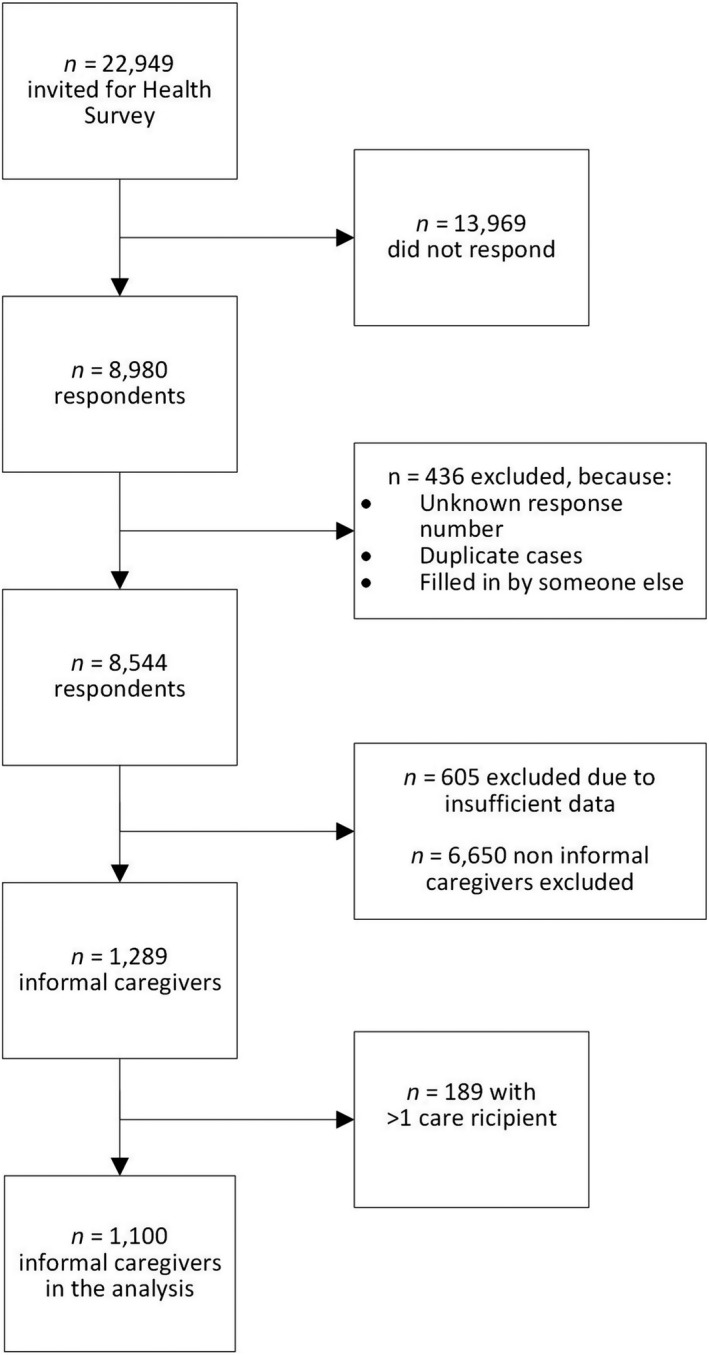
Flow chart of the study population

Of the 22,949 randomly selected adults by the Public Health Services of Zaanstreek‐Waterland and Statistics Netherlands, 8,980 (~39%) filled out the Health Survey. Approximately 5% of the participants were excluded for various reasons (as described in more detail in Figure 1), leaving 8,544 participants of whom 1,289 were informal caregivers that were eligible for the current study. For the regression analysis we included 1,100 informal caregivers (Table 2) caring for only one care recipient type; 1,029 of these informal caregivers had no missing data on any of the variables of the final model.

**TABLE 2:** Characteristics of adult informal caregivers included in the analysis, stratified by non‐burdened and burdened informal caregivers

*n* = 1,100	Non‐burdened informal caregivers *n* = 932 (85%)	Burdened informal caregivers *n* = 168 (15%)
	*n* (%)	*n* (%)
*Demographic*		
Gender		
Male	295 (85)	53 (15)
Female	637 (85)	115 (15)
Age		
19–34 years	71 (83)	15 (17)
35–49 years	223 (82)	51 (19)
50–64 years	638 (86)	102 (14)
Marital status		
Not married/not partnered	192 (83)	40 (17)
Married/partnered	733 (85)	126 (15)
Educational level		
Primary	258 (87)	40 (13)
Secondary	358 (83)	71 (17)
Tertiary	297 (85)	52 (15)
Paid work		
Not ≥ 20 hr	348 (83)	70 (17)
≥20 hr	563 (86)	94 (14)
Household composition		
No single parent family	860 (85)	148 (15)
Single parent family	71 (80)	18 (20)
*Caregiver situation*		
Time spent per week		
1–2 hr	339 (94)	20 (6)
3–4 hr	230 (90)	26 (10)
5–8 hr	186 (84)	35 (16)
9–16 hr	98 (70)	42 (30)
>16 hr	63 (60)	42 (40)
Relationship with care recipient		
Not child^a^	869 (87)	130 (13)
Child^a^	63 (62)	38 (38)
*Health‐related*		
Perceived health		
Moderate, poor or very poor	184 (74)	64 (26)
(Very) good	747 (88)	104 (12)
Chronic disease		
No chronic disease	584 (88)	81 (12)
Chronic disease	343 (80)	86 (20)
Mental health		
No high risk of anxiety or depression	887 (86)	142 (14)
High risk of anxiety or depression	43 (63)	25 (37)
*Socio‐financial*		
Loneliness		
Not lonely	612 (89)	73 (11)
Moderate to severe loneliness	313 (77)	93 (23)
Financial difficulties		
No financial difficulties	755 (87)	112 (13)
Some/great financial difficulties	155 (76)	50 (24)
Mastery of life		
Insufficient	65 (66)	33 (34)
Moderate	626 (85)	107 (15)
High	216 (90)	23 (10)
Contact neighbours		
Weekly or more	711 (85)	125 (15)
Less than weekly	112 (83)	42 (17)
Social cohesion neighbourhood		
Getting (very) well along together	253 (79)	66 (21)
Not getting (very) well along together	669 (87)	99 (13)

a Cases where informal caregivers filled in more than one option for care recipient type were not taken into regression analysis. Number of respondents might vary between variables due to item non‐response.

Descriptive characteristics of the total Health Survey sample (*n* = 8,544) of whom 1,289 (16%) adult informal caregivers were presented in Table 1. The majority of the adult informal caregivers (69%) was female. Of the adult informal caregivers, 40% were unemployed or providing less than 20 hr of paid work. The majority of adult informal caregivers (57%) reported that they provided ≤4 hr of informal care per week, with 9% reporting >16 hr of informal care per week. The proportion of informal caregivers who provided informal care to their child(ren) was 9%. Of the adult informal caregivers, 38% had a chronic disease themselves and 15% reported to be burdened.

### 3.2 Univariate and multivariate regression analyses

Table 3 shows results of univariate and multivariate regression analyses. Due to their correlation with the variables ‘chronic disease’ and ‘relationship with care recipient’, the variables ‘providing informal care for at least three months’, ‘being limited in activities due to chronic disease’ and ‘travel distance to care recipient’, were not included in this study, and therefore not presented in the tables. Univariate analyses indicated that none of the demographic factors were significantly (*p* < .2) associated with caregiver burden. Educational level (OR [95% CI]: 1.28 [0.84–1.95]) and household composition (OR: 1.47 [0.85–2.54]) tended to be associated with caregiver burden, but this association was not statistically significant. Of caregiving situation factors, time per week spent providing care and relationship with care recipient (child) were significantly associated with caregiver burden. Moreover univariate analyses indicated that all health‐related factors, thus perceived health, having a chronic disease and high risk on anxiety and depression, were significantly associated to heavy caregiver burden. Also all socio‐financial factors were significantly associated to heavy caregiver burden, except for contact with neighbours.

**TABLE 3:** Logistic regression with potential predictors of caregiver burden among adult informal caregivers

*N* = 1,100	Univariate model^a^ OR (95% CI)	Intermediate model^a^ OR (95% CI)	Final model^b^ OR (95% CI)
	*p < .2 **p < .1	*p < .2 **p < .1	*p < .1 **p < .05
Demographic			
Gender			
Male	ref		
Female	1.01 [0.71 – 1.43]		
Age			
19–34 years	ref		
35–49 years	1.08 [0.57 – 2.04]		
50–64 years	0.76 [0.42 – 1.37]		
Marital status			
Not married/partnered	ref		
Married/partnered	0.83 [0.56 – 1.22]		
Educational level			
Primary	ref		
Secondary	1.28 [0.84 – 1.95]		
Tertiary	1.13 [0.72 – 1.76]		
Employment status			
Not paid work (≥ 20 hr)	ref		
Paid work (≥ 20 hr)	0.83 [0.59 – 1.16]		
Household composition			
No single parent family	ref		
Single parent family	1.47 [0.85 – 2.54]		
Caregiving situation			
Time spent per week			
1–2 hr	ref	ref	ref
3–4 hr	1.92 [1.05 – 3.51]**	1.89 [1.03 – 3.47]**	1.74 [0.93 – 3.23]*
5–8 hr	3.19 [1.79 – 5.68]**	3.09 [1.73 – 5.51]**	2.78 [1.54 – 5.01]**
9–16 hr	7.26 [4.08 – 12.95]**	6.61 [3.69 – 11.85]**	5.41 [2.96 – 9.88]**
>16 hr	11.30 [6.22 – 20.52]**	8.68 [4.66 – 16.17]**	7.52 [3.93 – 14.39]**
Relationship with care recipient			
Not child	ref	ref	ref
Child	4.03 [2.59 – 6.28]**	2.24 [1.37 – 3.66]**	2.55 [1.51 – 4.31]**
Health‐related			
Perceived health			
Moderate, poor or very poor	2.50 [1.76 – 3.55]** ref	1.78 [1.15 – 2.76]**	1.80 [1.20 – 2.68]**
(Very) good		ref	ref
Chronic disease			
No chronic disease		ref	
Chronic disease	ref	1.32 [0.89 – 1.95]*	
Mental health	1.81 [1.30 – 2.52]**		
No high risk of anxiety or depression	ref	ref	
High risk of anxiety or depression	3.63 [2.15 – 6.13]**	2.45 [1.38 – 4.37]**	
Socio‐financial			
Loneliness			
Not lonely	ref	ref	ref
Moderate to severe loneliness	2.49 [1.78 – 3.48]**	1.93 [1.32 – 2.81]**	2.05 [1.41 – 2.99]**
Financial difficulties			
No financial difficulties	ref	ref	
Some/great financial difficulties	2.18 [1.49 – 3.17]**	1.31 [0.85 – 2.01]	
Mastery of own life			
Inadequate	4.77 [2.62 – 8.69]**	2.94 [1.48 – 5.82]**	
Mediocre	1.61 [1.00 – 2.59]**	1.34 [0.81 – 2.24]	
High	ref	ref	
Contact neighbours			
Weekly or more	ref		
Less than weekly	1.13 [0.77 – 1.65]		
Social cohesion neighbourhood			
Getting (very) well along	ref	ref	
Together not getting (very) well along together	1.76 [1.25 – 2.49]**	1.37 [0.94–1.98]*	

a Significant factors of the univariate model (*p* < .20) were included in the intermediate model.

b The most significant factors of the intermediate model were included in the final model *p* < .1.

In the final multivariate model, the time spent providing care, for example informal caregivers who provide care for >16 hr compared to those who provide 1–2 hr of care per week (OR: 7.52 [3.93–14.39]), and providing care to their own child(ren) (OR: 2.55 [1.51–4.31]) were associated with heavy caregiver burden. Moreover perceived health (OR: 1.80 [1.20–2.68]) and loneliness of the informal caregiver (OR: 2.05; 1.41–2.99) were associated with heavy caregiver burden. The final multivariate model showed a *R* ^2^ value (Nagelkerke) of .21, depicting 21% explained variance.

## 4 DISCUSSION

In this cross‐sectional study, we aimed to identify factors associated with caregiver burden in adult informal caregivers. The time providing informal care, proving informal care to their own child(ren), poor perceived health and loneliness of the informal caregiver were retained in the multivariate model and were shown to be statistically significantly associated with caregiver burden. To support the informal caregivers, policy and interventions should be developed that address these factors, for example reducing the number of hours providing care, supporting parents who care for their child(ren), maintaining the health and targeting loneliness of informal caregivers.

Our results of an association between time spent providing care and caregiver burden are in line with previous research (Karakis et al., 2014; Oliva‐Moreno et al., 2018; Piran et al., 2017; Prevo et al., 2018; Schulz & Sherwood, 2008; Tadema & Vlaskamp, 2010). For example, Kenny, King, and Hall (2014) found that providing informal care for ≥20 hr per week was associated with worse physical and mental health (Kenny et al., 2014). Another study showed that the capacity of informal caregivers to continue to care and to pursue other activities, such as work and leisure activities, can be disrupted when providing too many hours of care (Scherpenzeel & Storms, 2016).

We also found higher caregiver burden among informal caregivers who provide informal care to their own child(ren), compared to informal caregivers who provide care to other care recipients. This confirms earlier research (Piran et al., 2017; Raina et al., 2005), showing that taking care for child(ren) with a chronic disease is a main challenge for involved parents (Tadema & Vlaskamp, 2010). Moreover providing such care may impact on the physical and psychological health of the informal caregivers and affect the whole family function (Raina et al., 2005). Our findings are in contrast, however, with earlier work showing that burden was highest among those providing care to partners (van Groenou, de Boer, & Iedema, 2013). It is important to note that the latter study was conducted among older (>55 years) adults, which could explain the different findings.

Our result of an association between poor perceived health and caregiver burden is in line with previous studies (Cormac & Tihanyi, 2006; Karakis et al., 2014; Rowe, McCrae, Campbell, Benito, & Cheng, 2008). We can, however, not infer on the causality of this association based on our cross‐sectional data. Poor perceived health can be a predictor as well as a consequence of providing informal care. For example, longitudinal studies have demonstrated that informal caregivers are at greater risk for developing mild hypertension and serious illness, compared to non‐caregivers (Shaw et al., 1999). In line with this vision, poor perceived health could be interpreted as a negative consequence of caregiving (Buyck et al., 2011). However, some informal caregivers might already have had poor health before they started to provide care, as there are indications that unhealthy persons will engage more often in providing informal care than healthy persons (Brouwer et al., 2004; Savage, Fisher, & Birch, 2007).

The current study confirms that there is more loneliness among burdened caregivers compared to non‐burdened caregivers (Beeson, 2003; Shah, Wadoo, & Latoo, 2010; Vasileiou et al., 2017). Due to our cross‐sectional design, loneliness can be both an antecedent and a consequence of providing care. Providing informal care has been shown to be a commonly perceived stressor (Bevan, 2015), which can increase the desire for a supportive social network (Clyburn, Stones, Hadjistavropoulos, & Tuokko, 2000). When the social network is insufficiently supportive, feelings of loneliness may arise (van den Broek & Grundy, 2018). There are indications that informal caregivers socially isolate themselves, due to limited time and energy to invest in maintaining other relationships (Rand, Malley, Netten, & Forder, 2015; van de Maat, Vermaas, & Kruijswijk, 2012). In the current study, we analysed informal caregivers who were 19–64 years of age. This group, compared to older caregivers, is more likely to engage in other societal participation activities, including work. The combination of caregiving and other societal participation activities could be an extra burden, but could also provide possibilities to reduce the burden of these caregivers. For example, the workplace setting of the caregiver may be an important support network to deal with loneliness and thereby caregiver burden.

Unlike earlier research, the current study did not find that demographic factors, such as gender, age, marital status, educational level, employment status and household composition, were associated with caregiver burden (Karakis et al., 2014; Kenny et al., 2014; McCullagh, Brigstocke, Donaldson, & Kalra, 2005; Piran et al., 2017; Prevo et al., 2018; Schrank et al., 2016). These previous studies were, however, limited to certain cultural groups, age groups (i.e. mainly older adults), educational level and patient groups with certain conditions. Moreover the current study is conducted with data after the legislation changes in the Netherlands.

### 4.1 Strengths and limitations

An important strength of this study is the use of a large‐scale sample of adults from the general population. It is the first study with data, after the decentralisation of the social domain in the Netherlands in 2015, examining the association of a range of demographic, caregiving situation, health‐related and socio‐financial factors with caregiver burden. Since many governments of countries also aim to decentralise care provision (Eurocarers, 2017), this study provides worthy results that could be used to support policy and intervention development. Moreover data of the regional Health Survey were used (Statistics Netherlands, 2017), with widely accepted and validated questionnaires and definitions that are used by public health services throughout the Netherlands, Statistics Netherlands and the RIVM.

Another strength of this study is that the prediction model development was carried out in three steps with conservative cut‐off points for *p*‐values (Huysmans et al., 2017). By this procedure, variables were not unintentionally removed from the model. Nevertheless, the final model, based on aforementioned four factors, explained a relatively small percentage of 21% of the variance of caregiver burden. Additional variables about the caregiving situation (such as caregiving tasks, number of care recipients and degree of dependency), social contacts with family or friends and specific chronic diseases of the care recipient could increase the explained variance (Prevo et al., 2018). Such variables were not incorporated in the Health Survey questionnaire and should thus be considered in future research and surveys.

Apart from aforementioned strengths, there were also some limitations of this study. With regard to the generalisability of this study, the Zaanstreek‐Waterland region has less inhabitants aged 20–35 years and less single‐parent families in the region, compared to in the rest of the Netherlands (Municipal Health Services, 2018a). However, the perceived health and loneliness among inhabitants of Zaanstreek‐Waterland is comparable to the average of the Netherlands (Municipal Health Services, 2018b). Because Dutch regions could differ between each other in several indicators, one should be cautious to generalise these results to all Dutch (and other) regions.

Moreover only adult inhabitants aged 19–65 years were included in the analysis and informal caregivers who provided care to more than one type of care recipient were excluded. Although no clear differences were found in caregiver burden between those who provided care to one compared to more than one care recipient, excluding this particular group of informal caregivers may have limited the generalisability of our findings. Due to the raising demands on caregivers, it is expected that informal caregivers might have to care more often for more than one recipient in the future.

Due to our cross‐sectional study design, causality cannot be obtained from our findings. Assessing causality in the association of factors with caregiver burden should therefore be a key focus of future research. Another potential limitation is that most variables used were categorised, which might have led to loss in variance. However, cut‐off scores used for this categorisation were mostly based on established methods that are used by public health services in the Netherlands.

### 4.2 Practical implications

The findings of the current study provide insights that could help early identification of informal caregivers who may experience caregiver burden. For reducing burden of informal caregivers, policy and interventions should focus on the amount of time spent providing informal care, informal caregivers providing care to their own child(ren), perceived health, and loneliness of the informal caregivers.

Facilities for ‘respite care’, a temporary or complete takeover of the care with the aim to reduce the burden on informal caregivers, could be improved. Respite care could reduce the number of hours providing informal care among informal caregivers and thereby provide a better ‘caring – life balance’ (Kenny et al., 2014). In addition, more leisure time could increase the time for social contacts which might reduce social isolation and loneliness (Vasileiou et al., 2017) and could provide the informal caregiver with the time to pay attention to their own health. To this end, availability, accessibility and transparency of respite care should be improved, for example by supporting informal caregivers in applying for respite care. Also task division (e.g. by using existing mobile applications) could be encouraged among family, friends and neighbours to help the informal caregiver with certain tasks and thereby reduce the number of hours they spent on providing informal care (Schrank et al., 2016).

To maintain the social network of the informal caregiver, it is also important that social contacts of the informal caregiver understand the impact of providing informal care. Interventions based on stimulating the social network of the informal caregivers might decrease loneliness (and withdrawal from society) among informal caregivers.

## 5 CONCLUSION

Time spent at providing informal care, providing informal care to their own child(ren), poor perceived health and loneliness of the informal caregiver were found to be associated with caregiver burden. Due to the rising demands on the healthcare system, the need for informal caregivers is likely to increase in coming years. To support the informal caregivers, policy and intervention programs should be developed that support informal caregivers by reducing the number of hours providing informal care, support parents who care for their child(ren), maintain the health and reduce loneliness among informal caregivers.

## CONFLICT OF INTEREST

The authors declare no conflicts of interest.
